# Dosage Compensation of X-Linked Muller Element F Genes but Not X-Linked Transgenes in the Australian Sheep Blowfly

**DOI:** 10.1371/journal.pone.0141544

**Published:** 2015-10-27

**Authors:** Rebecca J. Linger, Esther J. Belikoff, Maxwell J. Scott

**Affiliations:** Department of Entomology, North Carolina State University, Campus Box 7613, Raleigh, NC, 27695–7613, United States of America; CNRS/University Lyon 1, FRANCE

## Abstract

In most animals that have X and Y sex chromosomes, chromosome-wide mechanisms are used to balance X-linked gene expression in males and females. In the fly *Drosophila melanogaster*, the dosage compensation mechanism also generally extends to X-linked transgenes. Over 70 transgenic lines of the Australian sheep blowfly *Lucilia cuprina* have been made as part of an effort to develop male-only strains for a genetic control program of this major pest of sheep. All lines carry a constitutively expressed fluorescent protein marker gene. In all 12 X-linked lines, female larvae show brighter fluorescence than male larvae, suggesting the marker gene is not dosage compensated. This has been confirmed by quantitative RT-PCR for selected lines. To determine if endogenous X-linked genes are dosage compensated, we isolated 8 genes that are orthologs of genes that are on the fourth chromosome in *D*. *melanogaster*. Recent evidence suggests that the *D*. *melanogaster* fourth chromosome, or Muller element F, is the ancestral X chromosome in Diptera that has reverted to an autosome in *Drosophila* species. We show by quantitative PCR of male and female DNA that 6 of the 8 linkage group F genes reside on the X chromosome in *L*. *cuprina*. The other two Muller element F genes were found to be autosomal in *L*. *cuprina*, whereas two Muller element B genes were found on the same region of the X chromosome as the *L*. *cuprina* orthologs of the *D*. *melanogaster Ephrin* and *gawky* genes. We find that the *L*. *cuprina* X chromosome genes are equally expressed in males and females (i.e., fully dosage compensated). Thus, unlike in *Drosophila*, it appears that the *Lucilia* dosage compensation system is specific for genes endogenous to the X chromosome and cannot be co-opted by recently arrived transgenes.

## Introduction

Very different mechanisms are used in the fly *Drosophila melanogaster*, the nematode *Caenorhabditis elegans* and in mammals to achieve X chromosome dosage compensation [1–3]. In *D*. *melanogaster* the male-specific lethal or MSL complex is essential for dosage compensation [4, 5]. In males, the MSL complex binds to active genes and upregulates expression, thereby achieving dosage compensation [6, 7]. The MSL complex is thought to initially bind to 150–300 high affinity or chromatin entry sites on the X chromosome and then spread to actively transcribed genes [4]. Thus most genes on the X chromosome do not need to bind the MSL complex with high affinity in order to be compensated. Consistent with this model is the finding that, in general, X-linked transgenes are dosage compensated [8–10]. For example, Kuroda and colleagues studied X-linked insertions of the autosomal *cg3702* and *Rpl40* genes [9]. Neither gene attracted the MSL complex at their endogenous position on chromosome 2. However, at X chromosome locations both the *cg3702* and *Rpl40* genes were bound by the MSL complex in males and dosage compensated.

**Fig 1:**
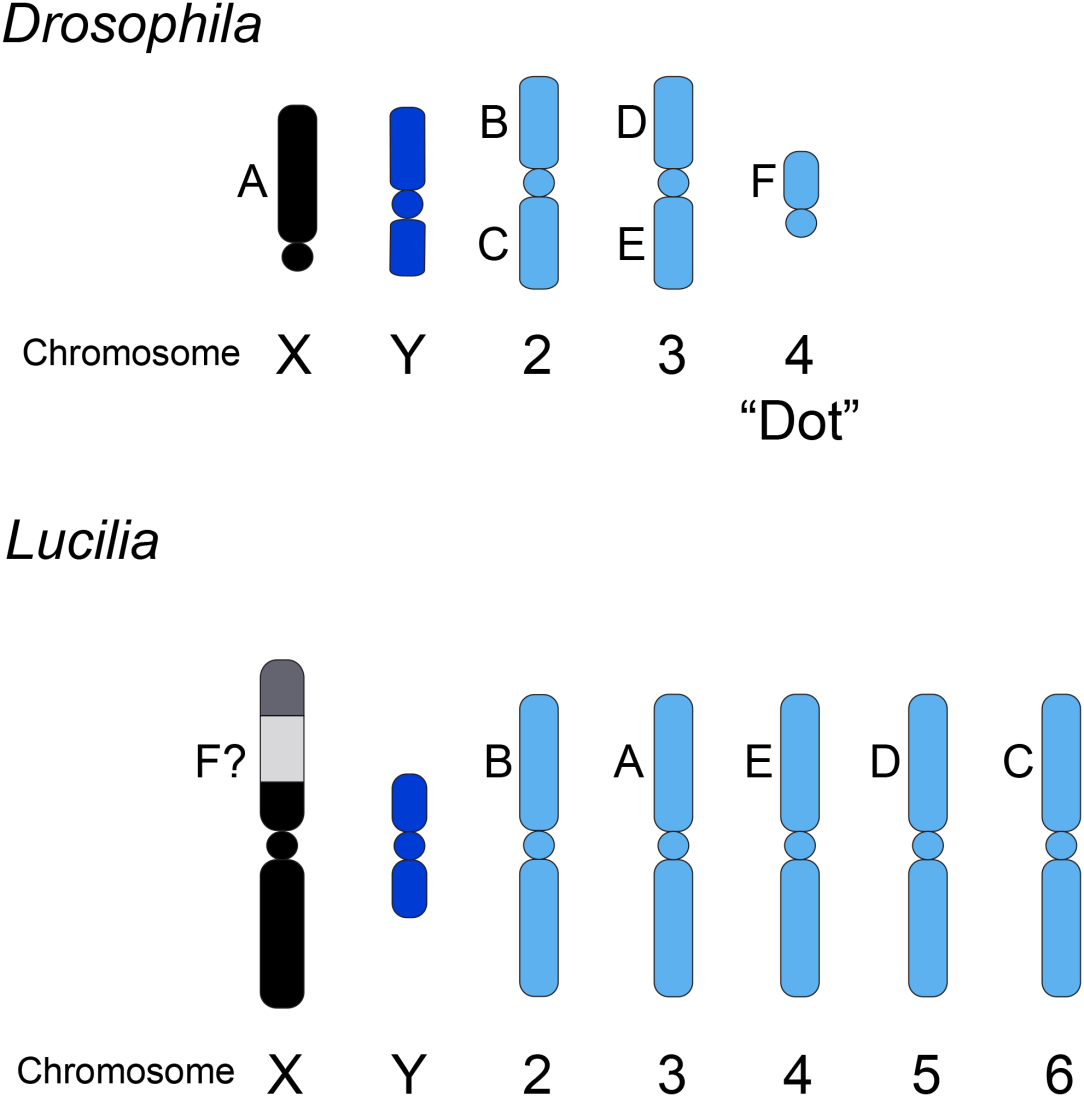
Schematic illustration of male metaphase chromosomes in *D*. *melanogaster* and *L*. *cuprina*. Muller elements (A-F) are indicated. The chromosomal location of linkage group F genes in *L*. *cuprina* was unclear from earlier genetic analysis but it has been suggested that these genes are on the X chromosome (this uncertainty is indicated by a question mark). In *L*. *cuprina*, C-banding produces dark staining of the sex chromosomes, except for a lighter staining region in the distal portion of the long arm of the X chromosome that is thought to contain active genes [13]. While the X chromosome is the largest chromosome, it is not drawn to scale so as to highlight the C-banding pattern.

The Australian sheep blowfly, *Lucilia cuprina*, is a major pest of sheep in Australia and New Zealand [11]. Since it is a serious pest, there was a significant effort to produce detailed physical and genetic maps of the chromosomes. *L*. *cuprina* has five pairs of metacentric autosomes of approximately equal size [12, 13]. C-banding of *L*. *cuprina* chromosomes from late third instar larval neuroblasts produces procentric bands on all autosomes and deep staining over the Y chromosome and most of the X chromosome [13]. The X chromosome is the longest chromosome and the Y is the shortest chromosome, less than half the length of the X chromosome. The distal third of the *L*. *cuprina* X chromosome is lighter staining and one morphological marker (black body, *b*) maps to this region (Fig 1) [13, 14]. Genetic analysis of 72 loci facilitated linkage group-chromosome assignments in *L*. *cuprina* [15] (Fig 1). The six linkage groups or Muller elements (A-F) have been generally conserved in higher Diptera. For example, orthologs of genes encoded by the right arm of chromosome 3 in *D*. *melanogaster* (linkage group E) were mapped to chromosome 4 in *L*. *cuprina* (Fig 1). A major difference is that the genes that are X-linked in *D*. *melanogaster* (linkage group A), are autosomal in *L*. *cuprina* and map to chromosome 3 (Fig 1). It has been shown that orthologs of genes located on the fourth chromosome in *D*. *melanogaster* (linkage group F), are X-linked in some non-drosophilids (e.g. tephritids) [16, 17]. It appears that the Muller element F was an ancestral X chromosome in Diptera but that in the lineage leading to *D*. *melanogaster* it has reverted to an autosome [16].

Over the past decade we have made over 70 transgenic *L*. *cuprina* lines as part of our effort to make male-only strains for a genetic control program [11, 18]. In general, the lines either express the tetracycline transactivator (tTA) or carry a tTA-regulated proapoptotic gene such *hid* or *reaper* [18, 19]. Twelve of the lines showed X-linked inheritance [20–23]. That is, when transgenic males were mated with virgin females from the parental wild type strain, only female offspring inherited the transgene. We were interested in determining if X-linked genes and transgenes were dosage compensated in *L*. *cuprina*. Our initial aim was to determine if the marker gene was dosage compensated in transgenic *L*. *cuprina* lines that map to the X chromosome. If X chromosome gene dosage compensation is achieved by a similar mechanism as in *D*. *melanogaster*, then it would be predicted that X-linked transgenes would be expressed equally in males and females in *L*. *cuprina*. With the goal of obtaining endogenous genes that are X-linked, our second aim was to identify *L*. *cuprina* genes that are orthologs of genes encoded by the fourth chromosome in *D*. *melanogaster* and determine if these genes were X-linked. If so, our third aim was to determine if the X-linked genes are expressed at equal levels in males and females (i.e. dosage compensated). We identified 8 orthologs of *Drosophila* fourth chromosome genes and show that 6 are located on the X chromosome in *L*. *cuprina* and fully dosage compensated. We also show that, unlike *Drosophila*, X-linked transgenes are not fully compensated. We suggest that this is because *L*. *cuprina* employs a novel dosage compensation mechanism.

## Results

### X-linked transgenes are not fully dosage compensated

**Fig 2:**
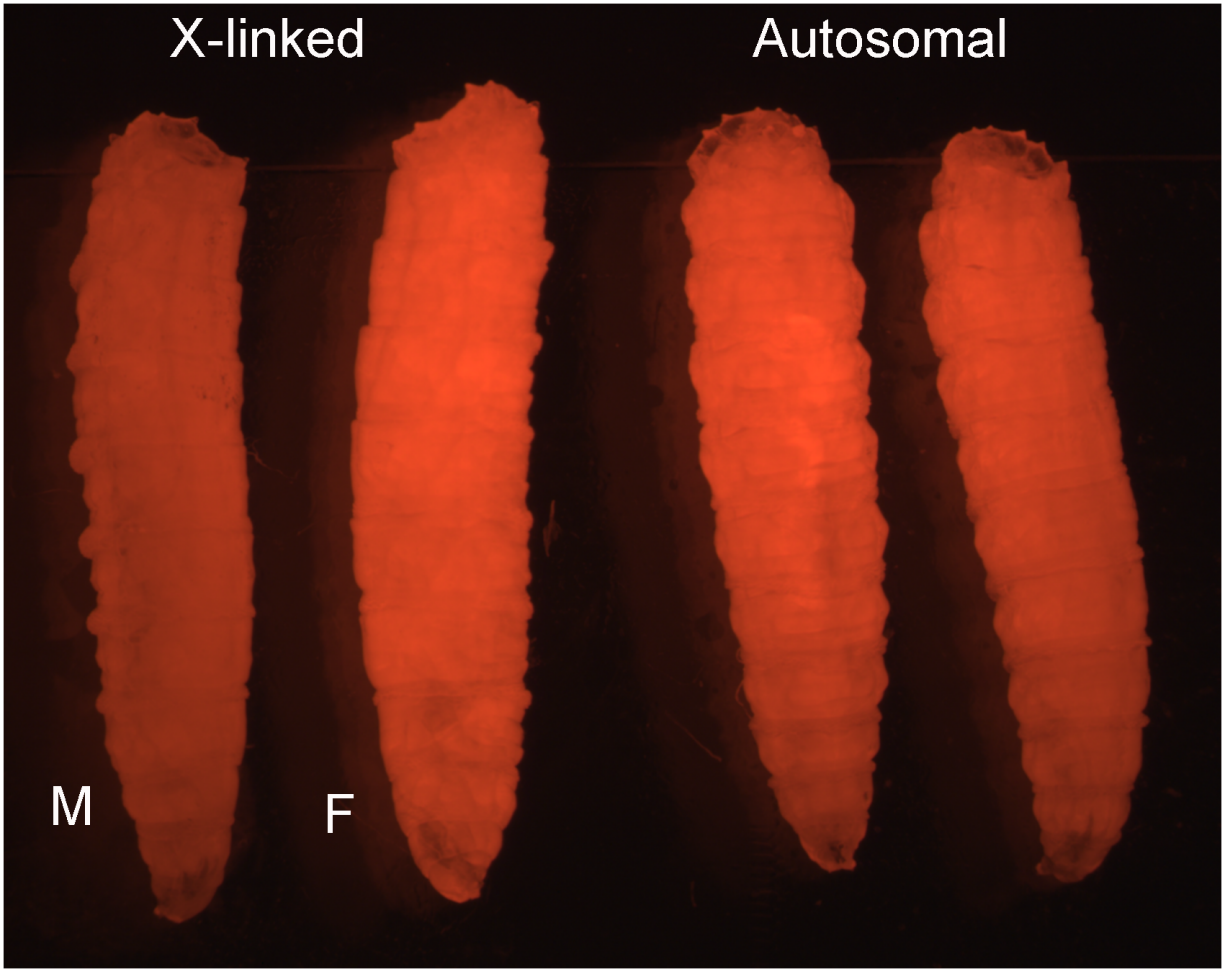
*L*. *cuprina* larvae with X-linked or autosomal fluorescent protein transgenes. The lines carry a constitutively expressed DsRed-express2 marker gene. Larvae from an X-linked line (SLAM5) and an autosomal line (EF3C) are shown. In the X-linked line, the most brightly fluorescent larvae develop as females (F). The more weakly fluorescent larvae develop as males (M). In contrast, larvae from the EF3C line show a uniform level of fluorescence. Two representative larvae from this line are shown, the sex of the larvae is unknown.

**Fig 3:**
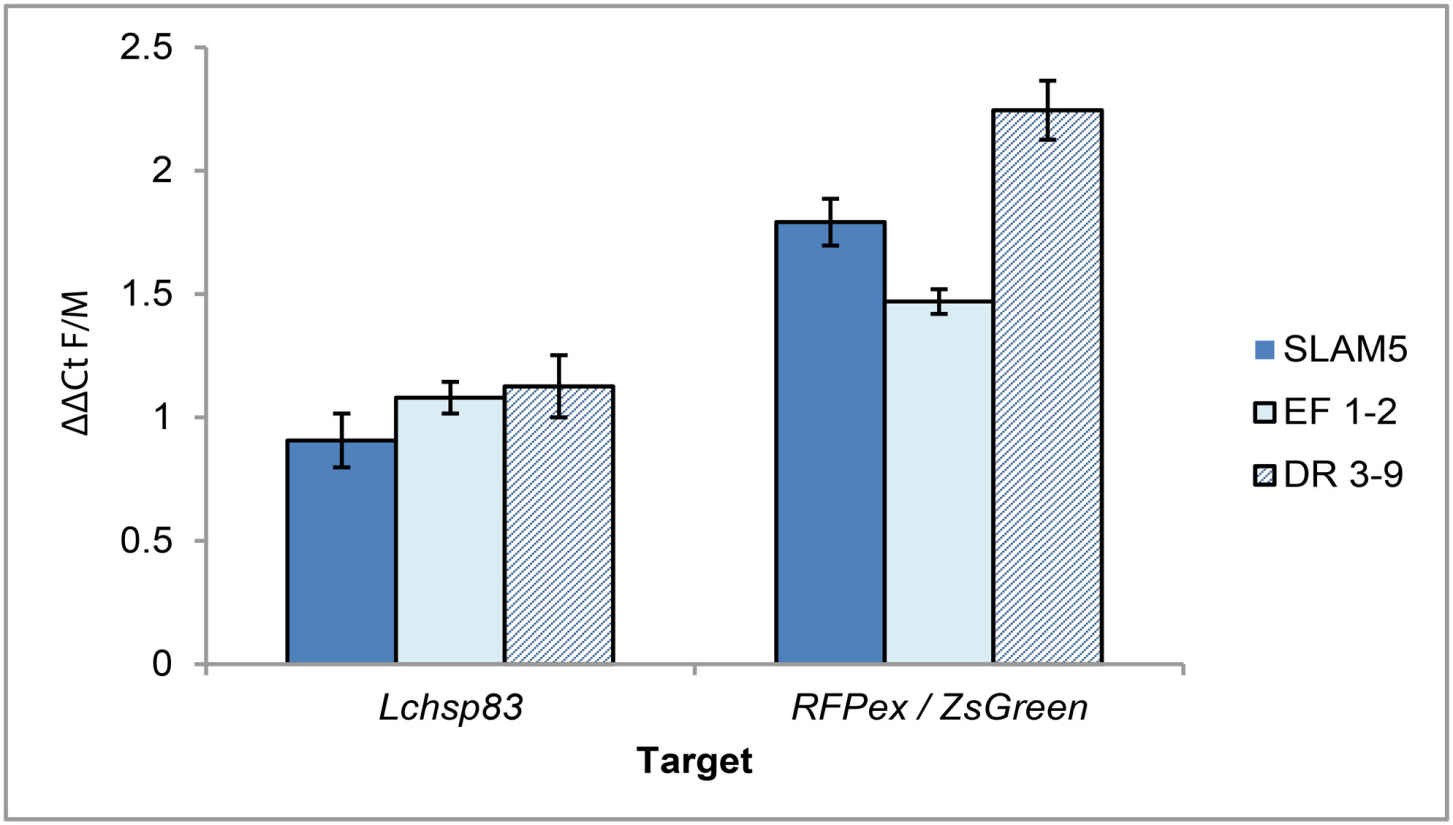
X-linked transgenes are not fully dosage compensated in *L*. *cuprina*. qRT-PCR of marker gene (ZsGreen or DsRed-express2) RNA in hemisected adult males and females from the transgenic lines SLAM5, EF 1–2 or DR3-9. All lines carry a single copy of the marker gene on the X chromosome. Transcript levels were normalized to 28S rRNA. As expression of the marker genes was driven by the constitutive promoter from the autosomal *Lchsp83* gene, *Lchsp83* transcript levels are shown for comparison. Mean female/male ratio +/- standard error from three biologically independent replicate experiments are shown.

The transgenic *L*.*cuprina* lines carry either a ZsGreen or DsRed-express2 (RFPex) fluorescent protein marker gene driven by the *L*. *cuprina hsp83* gene promoter that has a high basal activity [24]. Further details on the marker gene and any additional transgene in the X-linked lines are listed in S1 Table. Lines were bred to homozygosity by selecting for very brightly fluorescing larvae. In the X-linked lines, all of the brightly fluorescing larvae that are selected to make a homozygous line uniformly developed as females (Fig 2). With insertions on autosomes, approximately half of the most brightly fluorescent larvae develop as females and half as males (Fig 2). The concordance between fluorescence intensity and sex is strongly suggestive that X-linked marker gene expression is not fully dosage compensated. We next used quantitative RT-PCR (qRT-PCR) to confirm a lack of uniformly complete compensation. Four lines, SLAM5, DR3-9, EF1-2 and FL3-3 were selected for these experiments. In two lines, (SLAM5 and DR3-9) marker gene expression was twice as high in females compared to males (Fig 3). Thus, transgene expression is not dosage compensated. In a third line (EF1-2), females had 1.5 times as much transcript as males, suggesting partial compensation. In a fourth line (FL3-3) ZsGreen transcript levels were highly variable and much higher in females than males (5–14 times higher, not shown in Fig 3). In this line, the transgene has inserted into highly repetitive DNA and the marker shows variable and variegated patterns of expression (not shown).

### Orthologs of some *D*. *melanogaster* fourth chromosome genes are X-linked in *L*. *cuprina*

**Fig 4:**
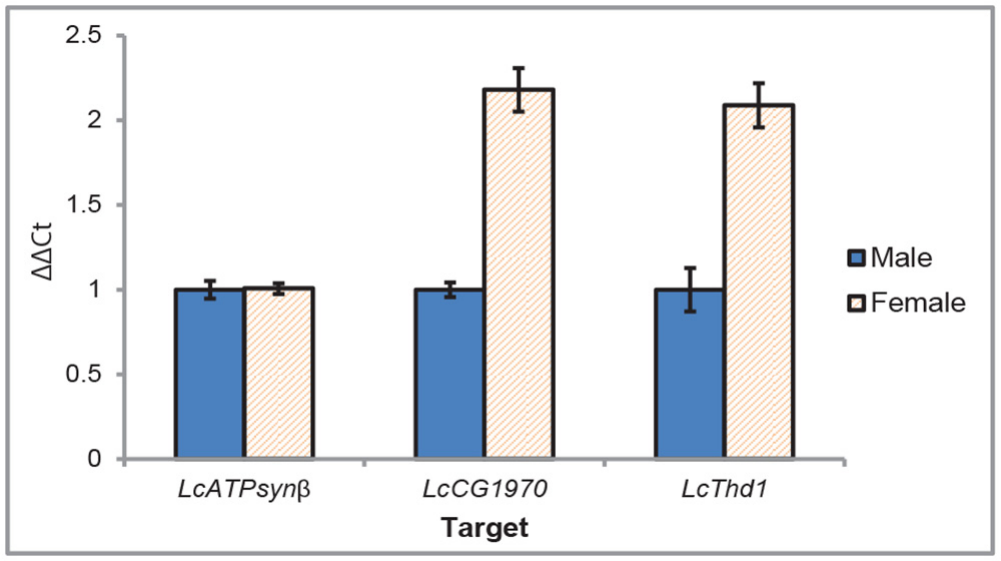
Linkage group F genes are X-linked in *L*. *cuprina*. qPCR of orthologs of *Drosophila* fourth chromosome genes with male and female *L*. *cuprina* genomic DNA. An X-linked gene is twice as abundant in female DNA relative to male DNA.

To investigate if linkage group F genes are X-linked in *L*. *cuprina*, we identified orthologs of *Drosophila* fourth chromosome genes in transcriptomes from the closely related blowfly, *Lucilia sericata* [25]. Most genes were identified from an embryo transcriptome, as this had the greatest depth of coverage. Using primers based on the *L*. *sericata* sequences, fragments of the homologous *L*. *cuprina* genes were amplified from genomic DNA, cloned, and sequenced. The genes isolated were the *L*. *cuprina* orthologs of, *ATP synthase-β* subunit (*ATPsynβ*), *CG1970*, *Eph* receptor tyrosine kinase (*Eph*), *Ephrin*, *Slip1*, *Thd1* and *Zn finger homeodomain 2* (*zfh2*). Quantitative PCR (qPCR) with male and female genomic DNA was used to determine X chromosome association. An X-linked gene would be twice as abundant in female relative to male DNA. In contrast, the copy number of an autosome gene would be the same in male and female DNA. Thus the expectation is that the male/female ratio of normalized Ct values would be either 0.5 or 1.0. For every gene, only one of the competing hypothetical ratios is contained by the calculated 95% confidence intervals (Table 1), so that there is little ambiguity as to whether or not the gene is sex-linked. We found that five of the *L*. *cuprina* (*Lc*) genes, *LcCG1970*, *LcEph*, *LcSlip1*, *LcThd1* and *Lczfh2* were associated with the X chromosome (Fig 4, Table 1). *LcATPsynβ* and *LcEphrin* are autosomal in *L*. *cuprina*. In addition, qPCR with male and female DNA confirmed the X-linked transgenes were associated with the X chromosome (Table 1). We also confirmed that the *L*. *cuprina* reference genes used in gene expression studies [26], *LcGST-1* and *Lchsp83* are autosomal (S2 Table). Further, males and females have equal levels of the 28S rRNA gene reference gene. As in *Drosophila*, rRNA genes are present on both the X and Y chromosomes in *L*. *cuprina* [27].

**Table 1:** Relative abundance of candidate X-linked genes and known X-linked transgenes in male and female genomic DNA.

Gene	ΔΔCt Male^d^± SEM	ΔΔCt Female± SEM	M/F Ratio (95% CI)	*p*-value M-F/2 = 0^e^	*p*-value M-F = 0^e^
*Lcaru* ^a^	1.0 ± 0.078	2.108 ± 0.129	0.475 (0.354–0.596)	0.616	0.00073
*LcATPsynβ* ^a^	1.0 ± 0.052	1.006 ± 0.031	0.994 (0.84–1.148)	0.00026	0.925
*LcCG1970* ^a^	1.0 ± 0.043	2.179 ± 0.128	0.459 (0.373-.0545)	0.298	0.00033
*LcEph* ^b^	1.0 ± 0.048	1.986 ± 0.092	0.503 (0.417–0.589)	0.92	0.00022
*LcEphrin* ^b^	1.0 ± 0.028	1.076 ± 0.038	0.930 (0.822–1.037)	0.00004	0.168
*Lcgw* ^a^	1.0 ± 0.047	2.284 ± 0.111	0.438 (0.362–0.514)	0.108	0.00013
*Lchsp83* ^c^	1.0 ± 0.017	0.953 ± 0.035	1.049 (0.94–1.158)	0.00001	0.281
*LcJwa*	1.0 ± 0.0549	2.08 ± 0.0506	0.481 (0.406–0.555)	0.5368	0.00003
*LcSlp1* ^b^	1.0 ± 0.066	2.127 ± 0.042	0.470 (0.386–0.553)	0.401	0.00003
*LcThd1* ^b^	1.0 ± 0.128	2.088 ± 0.130	0.479 (0.304–0.654)	0.771	0.0019
*Lczfh2* ^b^	1.0 ± 0.071	2.030 ± 0.053	0.493 (0.397–0.589)	0.85	0.00008
*RFPex* ^b^	1.0 ± 0.062	1.775 ± 0.036	0.563 (0.468–0.657)	0.142	0.00012
*ZsGreen* ^c^	1.0 ± 0.039	1.938 ± 0.111	0.516 (0.424–0.608)	0.667	0.0005

^a,b,c^Superscript indicates strain that was the source of genomic DNA for analysis.

^a^: wild type,

^b^: SLAM5, and

^c^: DR3-9.

^d.^ For each gene the male sample was chosen as the control/ calibrator sample and set to equal 1.0.

^e.^ Two *p*-values for each gene were calculated from the competing hypotheses that either the Male-Female/2 difference was zero or that the Male-female difference was zero. The latter would be expected for a M/F ratio of one.

### X-linked F-element genes are fully dosage compensated

qRT-PCR was used to determine if the X-linked genes were dosage compensated in *L*. *cuprina*. Young adults (1–4 day) were hemisected and RNA was isolated from heads and thoraces. Abdomens were discarded as they contain most of the sexually dimorphic tissues. If an X-linked gene is fully dosage compensated, the expectation is that males and females would express equal levels of mRNA transcripts. If, however, expression was not compensated then females would express twice the level of RNA than males. Student's t-tests were performed for each gene separately to investigate the hypothesis that male/female ratios were one. We found no evidence for departure from unity. That is, the five genes initially identified as on the X chromosome, *LcCG1970*, *LcEph*, *LcSlip1*, *LcThd1* and *Lczfh2* are all fully dosage compensated (Table 2).

**Table 2:** Dosage compensation of X chromosome genes in *L*. *cuprina*

Gene	Relative Expression Male:Female ± SEM ^a^	*p*-value M/F ratio ≠ 1^b^
*Lcaru* ^c^	1.179 ± 0.067	0.116
*LcCG1970*	0.967 ± 0.060	0.799
*LcEph*	0.975 ± 0.058	0.866
*Lcgw*	1.164 ± 0.132	0.34
*LcSlip1*	0.999 ± 0.068	0.992
*LcThd1*	0.887 ± 0.120	0.297
*Lczfh2*	0.998 ± 0.076	0.981

^a.^ Mean of 3 independent experiments. Expression was normalized to the 28S rRNA reference gene.

^b.^ Student's t-tests were performed for each gene separately

^c.^ With the exception of *Lcaru*, all genes are orthologs of genes located on the fourth chromosome in *D*. *melanogaster*. The *D*. *melanogaster aru* gene is located on chromosome 2.

### X chromosome genes do not appear to be highly clustered

**Fig 5:**
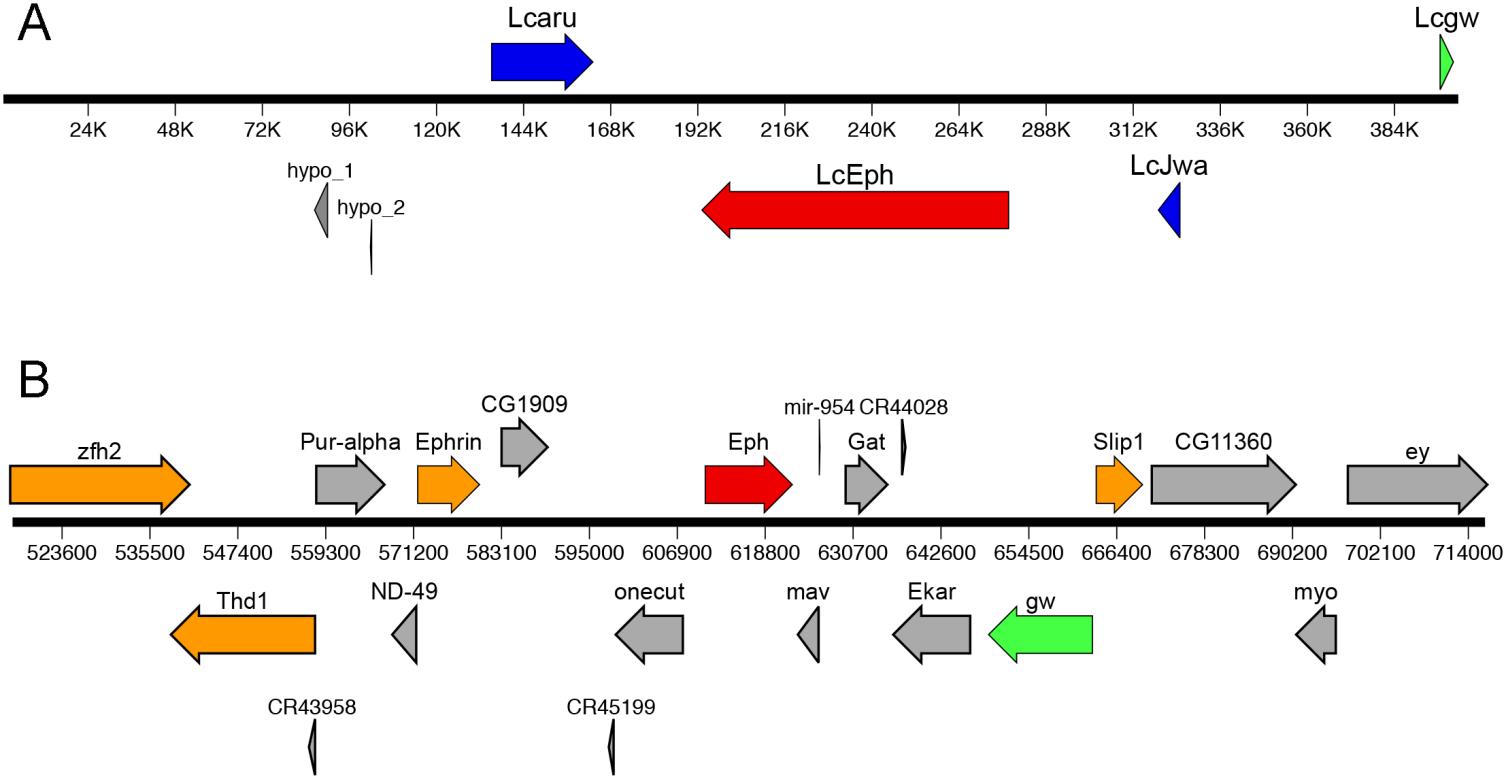
A comparison of the *Eph* genetic region in *L*. *cuprina* and *D*. *melanogaster*. The length and direction of arrows indicates the sizes of genes and direction of transcription. Genes in the *L*. *cuprina* 402 kb scaffold 328 (A) and 200 kb region of the *D*. *melanogaster* fourth chromosome containing the *Eph* gene (B) are shown. To facilitate comparison, *LcEph* and *Eph* are shown with red arrows whereas *Lcgw* and *gw* are in green. The *D*. *melanogaster* orthologs of *L*. *cuprina* genes that were part of this study are in orange. The *Lcaru* and *LcJwa* genes are in blue. The orthologous *aru* and *Jwa* genes are located on chromosome 2L in *D*. *melanogaster*. The approximate gene boundaries shown for the *L*. *cuprina* genes are based on alignments of transcripts with the genome sequence but remain to be experimentally confirmed.

One explanation for our results is that the fully compensated genes are clustered within a small region of the large X chromosome and that by chance none of the transgenes are within this compensated chromatin domain. Subsequent to our isolation of *L*. *cuprina* linkage group F genes, a draft assembly of the *L*. *cuprina* genome became available as part of the i5k project [28, 29]. All five genes we identified as on the X chromosome mapped to different scaffolds of the *L*. *cuprina* genome. The scaffold sizes containing X-linked genes ranged from 30 to 904 kb, with an average of size of 357 kb. As the assembly is at a draft stage, a comprehensive analysis of X chromosome genomic regions is premature. Nevertheless, we have analyzed the scaffolds containing the five genes in greater detail. The *L*. *cuprina* orthologs of the *D*. *melanogaster* fourth chromosome genes *CG1909* and *maverick* (*mav*) were found on the same scaffold (#114) that contained *LcCG1970*. Similarly part of the ortholog of the *Drosophila gawky* (*gw*) gene, *Lcgw*, was found on the same scaffold (#328) as *LcEph*. The other part of the *Lcgw* gene was found on a different scaffold (#1414). Orthologs of *D*. *melanogaster* fourth chromosome genes were not found on the scaffolds that contained the *LcSlip1*, *LcThd1* or *Lczfh2* genes. With the exception of the *Lczfh2* gene, the *Lucilia* X-linked genes are larger than their corresponding *Drosophila* ortholog (at least twice the size). To compare the *Drosophila* and *Lucilia Eph* chromosomal regions, the 402kb scaffold 328 was examined in greater detail. Genes were identified by searching for homology to *L*. *sericata* transcriptomes from different life stages [25]. In addition to the *LcEph* and *Lcgw* genes, we found the *L*. *cuprina* orthologs of the *arouser* (*aru*) and *Jwa* genes in scaffold 328 (Fig 5A). The *L*. *cuprina* genome project identified two additional genes in scaffold 328 that code for novel small hypothetical proteins (Fig 5A). We did not examine these genes further as transcripts were not found in the *L*. *sericata* transcriptomes and the no orthologous genes were identified in the *D*. *melanogaster* genome. In contrast to the few genes present in the scaffold containing the *LcEph* gene, over 20 protein-coding genes are located in a 200 kb region that contains *Eph* in *D*. *melanogaster* [30] (Fig 5B). Interestingly, the *D*. *melanogaster aru* and *Jwa* genes are not located on the fourth chromosome but are found on the left arm of chromosome 2 (linkage group B). The *LcEph*, *Lcaru* and *LcJwa* genes are 3–5 times larger than their corresponding *Drosophila* orthologs, due to an increase in size and number of introns. We confirmed that the *Lcgw*, *Lcaru* and *LcJwa* genes were associated with the X chromosome by qPCR with male and female genomic DNA (Table 1). In addition, qRT-PCR of male and female RNA from hemisected adults showed that the *Lcgw* and *Lcaru* genes are fully compensated (Table 2).

## Discussion

In the Australian sheep blowfly *L*. *cuprina*, the X chromosome is the largest chromosome and is mostly heterochromatic except for the lighter staining distal portion of the long arm [13]. Since *L*. *cuprina* is a major economic pest, a large effort was made to develop accurate genetic maps based on over 70 morphological and enzymatic markers [15]. Despite the relative ease of identifying X-linked mutations, only one gene was previously mapped to the X chromosome [14]. This suggested that the X chromosome had few genes compared to the autosomes. There are about 100 genes on the fourth chromosome in *Drosophila* species [31]. Thus, the apparent low number of X-linked genes in *L*. *cuprina* is consistent with the hypothesis that the X chromosome in non-drosophilid higher Diptera corresponds to the fourth chromosome in *Drosophila* [16]. Our study supports this hypothesis as we found that six of eight orthologs of *Drosophila* fourth chromosome genes were X-linked in *L*. *cuprina*. Consistent with our findings, it has recently been reported that the X chromosome in *L*. *sericata* contains linkage group F genes [32]. As the X chromosome accounts for 12.3% of the 570Mb male genome [33], this gives a size of 70Mb, much larger than the 4.4Mb *D*. *melanogaster* fourth chromosome. The distal 1.2Mb of the long arm of the *D*. *melanogaster* fourth chromosome is polytenized in salivary glands with 80 genes, a similar gene density as autosomes [31]. Similarly, it is possible that the active genes are located within the lighter staining region of the *L*. *cuprina* X chromosome. However, it would appear that gene density would be lower on the *L*. *cuprina* X chromosome than the *Drosophila* fourth chromosome. Consistent with this suggestion, the X-linked genes all mapped to a different scaffold in a draft assembly of the *L*. *cuprina* genome. Further, an analysis of a 400 kb scaffold containing the *LcEph* gene found far fewer protein-coding genes than are present in the region containing the *Eph* gene in *D*. *melanogaster*. Most of the *Lucilia* X-linked genes were larger than the corresponding orthologous *Drosophila* genes. For example, at approximately 85 kb, the *LcEph* gene is more than 7 times longer than the *D*. *melanogaster Eph* gene. However, the mean gene length for a protein-coding gene in *L*. *cuprina* is 12.197 kb [28], whereas the mean size is 6.935 kb in *D*. *melanogaster* [34]. Thus, it is not clear if the larger gene size of the few *Lucilia* X-linked genes we have studied is a general feature of the X chromosome or simply reflects that, on average, genes are longer in *Lucilia* than in *Drosophila*. Interestingly, two of the four protein-coding genes on the *LcEph* scaffold, *Lcaru* and *LcJwa*, are not orthologs of *D*. *melanogaster* fourth chromosome genes. Rather, the *aru* and *Jwa* genes are on 2L in *D*. *melanogaster*. So it is not only linkage group F genes that are on the X chromosome in *L*. *cuprina*. A more complete genome assembly will facilitate further analysis of X chromosome organization in *L*. *cuprina*.

An early study of radiation-induced deletion mutants suggested that X-linked genes were at least partially compensated in *L*. *cuprina* [14]. The deletion mutants contained the *black body* (*b*) gene, the one known X-linked morphological marker. Several of the X chromosome deficiencies uncovered vital genes linked to *b*. Further, females heterozygous for these deletions showed a characteristic phenotype of small body size, wing vein irregularities and variable fertility. Thus the lighter staining distal third of the X chromosome contains vital genes, which appear to be at least partially dosage compensated. We find that endogenous X-linked genes are fully compensated. In *Drosophila*, the male specific lethal (MSL) complex is required for X chromosome dosage compensation [4]. Somewhat surprisingly, *Drosophila* appears to employ an additional balancing mechanism to regulate gene expression of fourth chromosome genes [35]. The existence of such a mechanism was initially suggested to explain the viability of flies that are haploid for chromosome 4 (4/0) [36]. The Painting of fourth (POF) protein binds almost exclusively to the euchromatic portion of the fourth chromosome [37]. HP1a co-localizes with POF and in addition binds to the heterochromatic pericentromeric regions of chromosome 4 [38]. Loss of POF results in a significant decrease in fourth chromosome gene expression, suggesting POF has a stimulatory effect [35]. In contrast, HP1a appears to have a repressive effect on fourth chromosome gene expression. Thus the balancing mechanism somehow involves an interplay of POF and HP1a. The importance of *pof* for fourth chromosome dosage compensation is highlighted by the observation that 4/0 flies that are homozygous for a *pof* null allele are not viable [35]. It has been suggested that this dosage compensation mechanism is an evolutionary holdover from a *Drosophila* ancestor that employed *pof* to regulate gene expression of X-linked genes [16]. If so, this suggests that *pof* may play an important role in X chromosome dosage compensation in *L*. *cuprina*. This hypothesis could be tested by making a deletion mutation of the *L*. *cuprina pof* gene using CRISPR/Cas9 technology [39].

X chromosome dosage compensation can be achieved by increasing gene expression in males or decreasing expression in females. The majority of evidence shows that the *Drosophila* MSL complex acts by increasing expression of X-linked genes in males. There is some evidence that dosage compensation in *L*. *cuprina* may also be achieved by increasing expression in males. *L*. *cuprina* pupal trichogen cells contain large banded polytene chromosomes that have been used to develop detailed chromosome maps for all autosomes [40]. However, the X chromosomes in these preparations does not polytenize [41]. A portion of the X chromosome forms a loose granular structure, which is thought to correspond to the lightly stained C banded region of the long arm. The granular structure is observed in nuclei from male and female pupae. However, in XXY male pupae, one of the X chromosomes does not form the granular structure but instead is polytenized [41]. It was suggested that the polytene structure was less favorable for gene expression than the loose granular structure. If so, the formation of the polytene structure may have been in response to an increase in gene expression from both X chromosomes in XXY males.

In *D*. *melanogaster*, X-linked transgenes are often dosage compensated [8, 9]. This has been interpreted as a consequence of how the MSL complex regulates the expression of genes on the X chromosome. The MSL complex is thought to initially bind to high affinity or chromatin entry sites and then spread in *cis* to active genes, which could involve recognition of modified histone tails via the chromodomain of MSL3 [4]. In this way active X-linked transgenes would also be regulated by the MSL complex. In contrast, we find that X-linked transgenes are generally not dosage compensated in *L*. *cuprina*. We suggest three possible explanations for these results. Firstly, the X chromosome dosage compensation system in *L*. *cuprina* could be optimized for endogenous genes, which reside in a largely heterochromatic X chromosome environment. There is evidence that the POF/HP1a balancing system may be specific for fourth chromosome genes in *D*. *melanogaster*. A large number of transgenic lines have been obtained with fourth chromosome insertions of a *white* (*w*) transgene [31]. In many of these lines, eyes show variegated pigmentation, suggesting the *w* transgene has inserted in a relatively repressive chromatin environment. Indeed, the transgenes had often inserted into regions with high levels of bound HP1 in S2 cells [37]. However, the locations of the silenced transgenes did not correlate with regions where the chromosome 4 genes were silenced or weakly expressed. Given the stimulatory role of POF, it would be anticipated that in those lines that show whole eye pigmentation, the *w* transgene has inserted within or near a region with high levels of bound POF. However, in lines that show a wild type red eye phenotype, the *w* transgene has integrated into regions with low levels of POF. These results suggest that the fourth chromosome genes respond to POF/HP1a in a way that transgenes cannot [31, 37]. An alternative explanation for our observations is that the *Lchsp83* promoter that is driving fluorescent protein gene expression in the transgenes, cannot respond to the X chromosome dosage compensation machinery. We previously showed that the nature of the gene promoter is important in determining if an X-linked transgene is dosage compensated in *D*. *melanogaster* [42]. A *lacZ* transgene driven by the constitutive *armadillo* (*arm*) promoter was fully dosage compensated at several locations on the X chromosome. In contrast, a *lacZ* transgene driven by the neuronal *GMR-hsp70* enhancer/promoter was not dosage compensated when integrated at the same X chromosome locations as the *arm-lacZ* transgene. However, the *D*. *pseudoobscura hsp83* gene was fully dosage compensated at X chromosome sites in *D*. *melanogaster* [43, 44]. Thus the *Drosophila hsp83* promoter does respond to the MSL complex. A third explanation for the lack of compensation of X-linked transgenes is that the dosage compensation mechanism acts regionally rather than chromosome-wide. If the active genes are confined to the lighter staining distal portion of the long arm of the X chromosome, it would not be necessary to have a chromosome-wide dosage compensation mechanism. Given the large size of the X chromosome, it is possible than none of the twelve X-linked transgenes have integrated into a compensated chromatin domain. That is, none of the transgenes may have integrated into the lighter staining region of the X chromosome. A test of this model would be to insert a transgene directly adjacent to a compensated X-linked gene using CRISPR/Cas9 technology.

Studies on X chromosome gene expression in *L*. *cuprina* could have a practical application for control of this major pest of sheep. *msl2* RNA is not translated in female *D*. *melanogaster* [45]. Expression of MSL2 protein in females delays development and reduces viability [46]. Thus it may be possible to employ the *L*. *cuprina* dosage compensation machinery to make a male-only strain, which is advantageous for a genetic control program [18].

## Materials and Methods

### Insect rearing

*L*. *cuprina* larvae were reared on ground meat (90% lean meat/ 10% fat) and adults fed water, sugar and protein-rich biscuit as described previously [20]. Typically, 50 g of meat was used for rearing 100 mg of first instar larvae. Tetracycline (100 μg/mL) was added to the larval and adult diet for the FL3#3 line [21]. The progeny of each cross was screened at the late embryo/early first instar larval for green or red fluorescence using a Leica M165FC microscope with the appropriate filter set (GFP2 filter [ex480/40, em LP510 nm], DsRed filter [ex545/25, em 595/50 nm]). Images were captured using a Leica DFC500 digital camera and images initially analyzed using the Leica LAS v4.0 application suite. For RNA isolation, adults were fed sugar and water after eclosion and collected at 1–4 days old. Flies were anesthesized with CO_2_, sorted by sex, and hemisected. Heads and thoraxes were snap frozen in liquid nitrogen and stored at -80°C until processing.

### Genomic DNA isolation

Five to 6 frozen head/thoraxes were ground to powder with a mortar and pestle under liquid nitrogen. Powder was dissolved in 4 mL STE buffer (50 mM Tris-HCl, pH 7.5, 100 mM NaCl, 10 mM EDTA, pH 8). Two hundred μL 10% SDS and 8μL RNase A (Cat# R4642 Sigma Aldrich St. Louis, Missouri) were added and samples were incubated at 56°C. After 30 min, Proteinase K (Cat# P2308 Sigma Aldrich) was added to 100 μg/mL and the sample was incubated overnight at 56°C. Three mL phenol:chloroform:isoamyl alcohol [25:24:1] (Cat#P2069, Sigma) was added and samples were rotated 10 min at room temperature (RT). Samples were then centrifuged 10 min at 3000 RPM at 4°C. The aqueous layer was transferred to a new tube. The extraction was repeated. One tenth volume 3M NaAcetate, pH 5.2 and 2 volumes cold 100% ethanol were added and the samples were inverted 2–3 times. The samples were incubated at -20°C for 1 h and centrifuged 30 min at 6000 RPM at 4°C. The supernatant was removed from the pellet and 1 mL cold 75% ethanol was added. The samples were centrifuged 10 min at 6000 RPM at 4°C. The supernatant was removed from the pellet and it was allowed to air dry 10 min. The last of the supernatant was removed and the pellet was allowed to air dry 10 additional minutes before being resuspended in 50–100 μL TE Buffer.

### Primer design and efficiency testing

First, primers were designed for a 300–600bp region of the largest exon of candidate X-linked genes identified in *L*. *sericata* transcriptomes [25]. Five hundred ng of genomic DNA from the *L*. *cuprina* LA07 wild type strain (parental strain for transgenic lines) was used as template for PCR with MangoTaq (Cat# BIO-21083 Bioline, London, UK). Cycling conditions were: (95°C 3 min, [95°C 30 sec, 55°C 30 sec, 72°C 1 min] 30x, 72°C 8 min). For some primer sets, PCR was performed using OneTaq Hot Start 2X Master Mix (Cat#M0484S New England Biolabs) (94°C 3 min, [94°C 15 sec, 55°C 30 sec, 68°C 1 min] 30x, 68°C 5 min). PCR products were resolved on a 1% agarose gel and band slices were removed. Bands were gel purified, cloned into pGEMT-Easy (Promega, Fitchburg, WI, USA) and sequenced. The resulting sequence was used to design qPCR primers for each gene candidate. Primers were tested for efficiency by creating a dilution series from 0.00005ng- 500ng template genomic DNA per well. Thermo Maxima SYBR Green/ Rox qPCR Master Mix 2X (Cat#K0221 Thermo Fisher Scientific Waltham, Massachusetts) was used with the indicated primers to create a master mix. Template was added in quadruplicate wells of a 384 well optical plate (Cat#4309849 Applied Biosystems/ Life Technologies/ Thermo Fisher Scientific Waltham, Massachusetts), followed by master mix, which was added by a multi-channel pipet. The plate was sealed (Cat#4311971 Applied Biosystems/ Life Technologies/ Thermo Fisher Scientific Waltham, Massachusetts), vortexed, centrifuged 1 min at RT at 3400 RPM, and run. The run was performed on a BioRad CFX384 C1000 Touch Thermocycler (BioRad Hercules, CA) using the following program: 95°C 10 min, [95°C 15 s, 60°C 60 sec] 40x. Data acquisition was performed on the anneal/ extension step.

Primer efficiency was determined by plotting the log of the ng of template on the X-axis and the mean Ct of the quadruplicate replicates on the Y-axis. The slope of the best fit line was used in the following equation to calculate efficiency: [Efficiency = -1+10^(-1/slope)^]. Primers were accepted if efficiency was 90–105% and re-designed if efficiency fell outside this range. The oligonucleotide primers used in this study are listed in S3 Table.

### RNA isolation

Two frozen head/thoraxes were homogenized in 500 μL of Trizol (Cat#15596026 Life Technologies/ Thermo Fisher Scientific Waltham, Massachusetts) in a 1 mL glass homogenizer that had previously been baked at 200°C overnight. 100 μL of chloroform was added, and samples were shaken for 15 s and allowed to incubate at RT for 15 min. Subsequently, samples were centrifuged at 13,000 RPM 15 minutes at 4°C. The aqueous layer was mixed with an equal volume cold RNase-free 70% ethanol, mixed vigorously, and loaded on onto a Qiagen RNeasy Mini Kit column (Cat#74104 Qiagen Venlo, Netherlands). DNAse treatment was performed on-column in accordance with the manufacturer’s recommended protocol using the RNase-free DNAse set (Cat#79254 Qiagen). To achieve complete elimination of DNA contamination, a second in-solution treatment was performed on 30 μg of total RNA using this set, followed by re-purification with the RNeasy mini kit according to manufacturer’s protocol.

### qRT-PCR

To synthesize cDNA, 3.5 μg of DNAse treated RNA was utilized with the Superscript III First Strand Synthesis Supermix (Cat#18080–400 Invitrogen/ Life Technologies/ Thermo Fisher Scientific Waltham, Massachusetts) according to kit protocol. Random hexamers were used as primers. Negative control reactions containing water instead of enzyme mix were performed to confirm the absence of DNA contamination.

The cDNA template was diluted 1:4 with nuclease-free water. Thermo Maxima SYBR Green/ Rox qPCR Master Mix 2X (Cat#K0221 Thermo Fisher Scientific Waltham, Massachusetts) was used with the indicated primers to create a master mix. Template was added in quadruplicate wells of a 384 well optical plate (Cat#4309849 Applied Biosystems/ Life Technologies/ Thermo Fisher Scientific Waltham, Massachusetts), followed by master mix, which was added by a multi-channel pipet. The plate was sealed (Cat#4311971 Applied Biosystems/ Life Technologies/ Thermo Fisher Scientific Waltham, Massachusetts), vortexed, centrifuged 1 min at RT at 3400 RPM and run. The run was performed on a BioRad CFX384 C1000 Touch Thermocycler (BioRad Hercules, CA) using the following program: 95°C 10 min, [95°C 15s, 60°C 60 sec] 40x. Data acquisition was performed on the anneal/ extension step.

Data analysis of delta delta Ct was performed using BioRad CFX Manager. Mean Ct value was found for the 4 replicate wells. *L*. *cuprina* 28S rRNA was previously determined to be a suitable reference gene by geNORM and Normfinder [47], and was therefore chosen as the reference gene for this study. 28S rRNA was also confirmed in our hands to have a similar expression in *L*. *cuprina* EF1-2 males and females as measured by the mean of quadruplicate Ct values (S2 Table). Delta delta Ct was calculated and the male sample was chosen as the control/calibrator sample and set to equal 1.0. Replicate bar graphs represent delta delta Ct, with error bars delineating standard error of the mean. To calculate summary ratio, the male delta delta Ct was divided by the female delta delta Ct. The 3 biological replicates were then averaged and the standard error found.

### qPCR

To confirm X-linkage, 5 ng of male or female genomic DNA was utilized as template and cycling conditions were as for qRT-PCR. *L*. *cuprina glutathione S-transferase 1* (*LcGST1*) was used as the reference gene. *LcGST1* was previously determined to be a suitable reference gene for qRT-PCR [47] and we found that the same primer pair efficiently amplified the correct DNA sequence from genomic DNA template. To confirm that *LcGST1* was autosomal, we performed qPCR with male and female genomic DNA from line EF1-2, which carries a single copy of a marker gene on the X chromosome. Male/female ratio of mean Ct values with the *LcGST1* primer pair from this line gave a ratio of 1.0, as expected (S2 Table). Analysis of delta delta Ct was performed using BioRad CFX Manager. Mean Ct value was found for the 4 replicate wells. The male sample was chosen as the control sample and set to 1. Samples with a male to female ratio close to 0.5 were determined to be X-linked.

### *L*. *cuprina* gene identification and annotation

To identify orthologs of *D*. *melanogaster* fourth chromosome genes, we performed tblastn searches [48] of *L*. *sericata* transcriptomes [25] with the *D*. *melanogaster* protein sequence. To confirm the correct ortholog had been identified, the *L*. *sericata* transcript was translated and a blastp search of annotated *D*. *melanogaster* protein database was performed. Gene boundaries were identified by blastn alignments of *L*. *sericata* transcripts with the *L*. *cuprina* genome [28]. From these alignments, exon-intron boundaries were identified, which generally matched the consensus sequences for 5' and 3' splice sites. For some genes, such as those belonging to multi-gene families, it was uncertain if the *Lucilia* gene that had been identified was the ortholog of the *D*. *melanogaster* fourth chromosome gene. Such genes were not further investigated.

### Statistical Analyses

#### qRT-PCR

To investigate the hypothesis that the Male/Female ratios of RNA are one, Student’s t-tests were performed for each gene separately based on the data from three or four independent replicates. No evidence of departure from unity was found. These tests suffer from low power, however, since they are based on only 3 or 4 replicates. As an alternative, we used a one-way factorial effects model (with PROC GLM in SAS) for the differences of the ratios from unity (ratio minus 1) and tested the hypothesis that all the underlying means for these differences were 0 for every gene. (H0: mu1 = mu2 =…= mu9 = 0) This enables us to pool information about variability in measurement of the ratios over all genes. The degrees of freedom for the tests for each gene go from 2 or 3 in the initial investigation up to 20 for the one-way ANOVA. There is very weak or no evidence of any departure of the ratios from 1. One gene, *Lcaru*, showed a *p*-value of less than 0.05 (0.0475) however, after Bonferroni adjustment for multiplicity of genes, the *p*-value was no longer significant.

#### qPCR

To determine whether the genes were autosomal or located on a sex chromosome, we used Fieller's method [49] to obtain approximate 95% confidence intervals for the gene-specific Male to Female ratios. In the construction of these intervals, we use multipliers from t-distributions with 5 degrees of freedom (2 for males, whose measurements have been normalized to unity and 3 for females.) Additionally, we computed two *p*-values for each gene from the competing hypotheses that either the Male-female difference was zero, or that the difference Male-Female/2 was zero based on corresponding t-statistics, again with 5 degrees of freedom.

## Supporting Information

S1 Table Transgenic lines of *Lucilia cuprina* that show X-linked inheritance. (DOCX)

S2 Table Abundance of reference genes in male and female genomic DNA. (DOCX)

S3 Table Primers utilized for qPCR. (DOCX)

S4 Table Raw Ct values for qPCR data shown in Fig 3. (XLSX)

S5 Table Raw Ct values for qPCR data shown in Fig 4. (XLSX)

S6 Table Raw Ct values for qPCR data shown in Table 1. (XLSX)

S7 Table Raw Ct values for qPCR data shown in Table 2. (XLSX)

## References

[1] Lucchesi JC. Dosage compensation in flies and worms: the ups and downs of X-chromosome regulation. Current Opinion in Genetics & Development. 1998;8(2):179–84. 9610408 10.1016/s0959-437x(98)80139-1

[2] Akhtar A. Dosage compensation: an intertwined world of RNA and chromatin remodelling. Curr Opin Genet Dev. 2003;13(2):161–9. . 12672493 10.1016/s0959-437x(03)00016-9

[3] Ferrari F, Alekseyenko AA, Park PJ, Kuroda MI. Transcriptional control of a whole chromosome: emerging models for dosage compensation. Nat Struct Mol Biol. 2014;21(2):118–25. 10.1038/nsmb.2763 24500429 PMC4342042

[4] Gelbart ME, Kuroda MI. *Drosophila* dosage compensation: a complex voyage to the X chromosome. Development. 2009;136(9):1399–410. 10.1242/dev.029645 19363150 PMC2674252

[5] Conrad T, Akhtar A. Dosage compensation in *Drosophila melanogaster*: epigenetic fine-tuning of chromosome-wide transcription. Nat Rev Genet. 2012;13(2):123–34. 10.1038/nrg3124 . 22251873

[6] Larschan E, Bishop EP, Kharchenko PV, Core LJ, Lis JT, Park PJ, et al X chromosome dosage compensation via enhanced transcriptional elongation in *Drosophila* . Nature. 2011;471(7336):115–8. Epub 2011/03/04. nature09757 [pii] 10.1038/nature09757 . 21368835 PMC3076316

[7] Conrad T, Cavalli FM, Vaquerizas JM, Luscombe NM, Akhtar A. *Drosophila* dosage compensation involves enhanced Pol II recruitment to male X-linked promoters. Science. 2012;337(6095):742–6. 10.1126/science.1221428 . 22821985

[8] Fitzsimons HL, Henry RA, Scott MJ. Development of an insulated reporter system to search for *cis*-acting DNA sequences required for dosage compensation in *Drosophila* . Genetica. 1999;105(3):215–26. 10761105 10.1023/a:1003801402153

[9] Gorchakov AA, Alekseyenko AA, Kharchenko P, Park PJ, Kuroda MI. Long-range spreading of dosage compensation in *Drosophila* captures transcribed autosomal genes inserted on X. Genes Dev. 2009;23(19):2266–71. Epub 2009/10/03. 23/19/2266 [pii] 10.1101/gad.1840409 . 19797766 PMC2758747

[10] Spradling AC, Rubin GM. The effect of chromosomal position on the expression of the *Drosophila xanthine dehydrogenase* gene. Cell. 1983;34(1):47–57. 6309411 10.1016/0092-8674(83)90135-6

[11] Sandeman RM, Levot GW, Heath AC, James PJ, Greeff JC, Scott MJ, et al Control of the sheep blowfly in Australia and New Zealand—are we there yet? Int J Parasitol. 2014;44(12):879–91. 10.1016/j.ijpara.2014.08.009 . 25240442

[12] Ullerich F-H. Geschlechtschromosomen und Geschlechtsbestimmung bei einigen Calliphorinen (Calliphoridae, Diptera). Chromosoma. 1963;14:45–110.

[13] Bedo DG. C, Q and H-banding in the analysis of Y chromosome rearrangements in *Lucilia cuprina* (Wiedemann) (Diptera: Calliphoridae). Chromosoma. 1980;77(3):299–308. 7189455 10.1007/BF00286055

[14] Maddern RH, Bedo DG. Properties of the sex chromosomes of *Lucilia cuprina* deduced from radiation studies. Genetica. 1984;63:203–12.

[15] Weller GL, Foster GG. Genetic maps of the sheep blowfly *Lucilia cuprina*: linkage-group correlations with other dipteran genera. Genome. 1993;36(3):495–506. 8349126 10.1139/g93-068

[16] Vicoso B, Bachtrog D. Reversal of an ancient sex chromosome to an autosome in *Drosophila* . Nature. 2013;499(7458):332–5. 10.1038/nature12235 23792562 PMC4120283

[17] Landeen EL, Presgraves DC. Evolution: from autosomes to sex chromosomes—and back. Curr Biol. 2013;23(18):R848–50. 10.1016/j.cub.2013.08.021 . 24070447

[18] Scott MJ. Development and evaluation of male-only strains of the Australian sheep blowfly, *Lucilia cuprina* . BMC Genet. 2014;15 Suppl 2:S3 10.1186/1471-2156-15-S2-S3 25472415 PMC4255793

[19] Heinrich JC, Scott MJ. A repressible female-specific lethal genetic system for making transgenic insect strains suitable for a sterile-release program. Proc Natl Acad Sci U S A. 2000;97(15):8229–32. 10890889 10.1073/pnas.140142697 PMC26929

[20] Concha C, Scott MJ. Sexual Development in *Lucilia cuprina* (Diptera, Calliphoridae) Is Controlled by the *Transformer* Gene. Genetics. 2009;182(3):785–98. Epub 2009/05/13. genetics.109.100982 [pii] 10.1534/genetics.109.100982 . 19433631 PMC2710159

[21] Li F, Wantuch HA, Linger RJ, Belikoff EJ, Scott MJ. Transgenic sexing system for genetic control of the Australian sheep blow fly *Lucilia cuprina* . Insect Biochem Mol Biol. 2014;51:80–8. 10.1016/j.ibmb.2014.06.001 . 24928635

[22] Edman RM, Linger RJ, Belikoff EJ, Li F, Sze SH, Tarone AM, et al Functional characterization of calliphorid cell death genes and cellularization gene promoters for controlling gene expression and cell viability in early embryos. Insect Mol Biol. 2015;24(1):58–70. 10.1111/imb.12135 . 25225046

[23] Yan Y, Scott MJ. A transgenic embryonic sexing system for the Australian sheep blow fly *Lucilia cuprina* . Sci Rep. 2015;in press. 10.1038/srep16090 PMC4633611 26537204

[24] Concha C, Belikoff EJ, Carey BL, Li F, Schiemann AH, Scott MJ. Efficient germ-line transformation of the economically important pest species *Lucilia cuprina* and *Lucilia sericata* (Diptera, Calliphoridae). Insect Biochemistry & Molecular Biology. 2011;41(1):70–5. Epub 2010/09/28. S0965-1748(10)00212-2 [pii] 10.1016/j.ibmb.2010.09.006 . 20869440

[25] Sze SH, Dunham JP, Carey B, Chang PL, Li F, Edman RM, et al A *de novo* transcriptome assembly of *Lucilia sericata* (Diptera: Calliphoridae) with predicted alternative splices, single nucleotide polymorphisms and transcript expression estimates. Insect Molecular Biology. 2012;21(2):205–21. Epub 2012/01/31. 10.1111/j.1365-2583.2011.01127.x . 22283785

[26] Concha C, Edman RM, Belikoff EJ, Schiemann AH, Carey B, Scott MJ. Organization and expression of the Australian sheep blowfly (*Lucilia cuprin*a) *hsp23*, *hsp24*, *hsp70* and *hsp83* genes. Insect Molecular Biology. 2012;21(2):169–80. Epub 2012/04/17. . 22506286 10.1111/j.1365-2583.2011.01123.x

[27] Bedo DG. Nucleolar fragmentation in polytene trichogen cells of *Lucilla cuprina* and *Chrysomya bezziana* (Diptera: Calliphoridae). Genome. 1992;35(2):283–93. 1618388 10.1139/g92-044

[28] Anstead CA, Korhonen PK, Young ND, Hall RS, Jex AR, Murali SC, et al *Lucilia cuprina* genome unlocks parasitic fly biology to underpin future interventions. Nat Commun. 2015;6:7344 10.1038/ncomms8344 26108605 PMC4491171

[29] Poelchau M, Childers C, Moore G, Tsavatapalli V, Evans J, Lee CY, et al The i5k Workspace@NAL—enabling genomic data access, visualization and curation of arthropod genomes. Nucleic Acids Res. 2015;43(Database issue):D714–9. 10.1093/nar/gku983 25332403 PMC4384035

[30] Pierre SE St, Ponting L, Stefancsik R, McQuilton P, FlyBase C. FlyBase 102—advanced approaches to interrogating FlyBase. Nucleic Acids Res. 2014;42(Database issue):D780–8. 10.1093/nar/gkt1092 24234449 PMC3964969

[31] Riddle NC, Shaffer CD, Elgin SC. A lot about a little dot—lessons learned from *Drosophila melanogaster* chromosome 4. Biochem Cell Biol. 2009;87(1):229–41. 10.1139/O08-119 19234537 PMC2950803

[32] Vicoso B, Bachtrog D. Numerous transitions of sex chromosomes in Diptera. PLoS Biol. 2015;13(4):e1002078 10.1371/journal.pbio.1002078 25879221 PMC4400102

[33] Ullerich FH, Schottke M. Karyotypes, constitutive heterochromatin, and genomic DNA values in the blowfly genera *Chrysomya*, *Lucilia*, and *Protophormia* (Diptera: Calliphoridae). Genome. 2006;49(6):584–97. Epub 2006/08/29. g06-013 [pii] 10.1139/g06-013 . 16936838

[34] Hoskins RA, Carlson JW, Wan KH, Park S, Mendez I, Galle SE, et al The Release 6 reference sequence of the *Drosophila melanogaster* genome. Genome Res. 2015;25(3):445–58. 10.1101/gr.185579.114 25589440 PMC4352887

[35] Johansson AM, Stenberg P, Bernhardsson C, Larsson J. Painting of fourth and chromosome-wide regulation of the 4th chromosome in *Drosophila melanogaster* . Embo J. 2007 . 17318176 10.1038/sj.emboj.7601604 PMC1864965

[36] Hochman B. The fourth chromosome of *Drosophila melanogaster* In: Ashburner M, Novitski E, editors. The genetics and biology of *Drosophila*. 1b. New York: Academic Press; 1976 p. 903–28.

[37] Johansson AM, Stenberg P, Pettersson F, Larsson J. POF and HP1 bind expressed exons, suggesting a balancing mechanism for gene regulation. PLoS Genet. 2007;3(11):e209 10.1371/journal.pgen.0030209 18020713 PMC2077892

[38] Riddle NC, Jung YL, Gu T, Alekseyenko AA, Asker D, Gui H, et al Enrichment of HP1a on *Drosophila* chromosome 4 genes creates an alternate chromatin structure critical for regulation in this heterochromatic domain. PLoS Genet. 2012;8(9):e1002954 10.1371/journal.pgen.1002954 23028361 PMC3447959

[39] Bassett A, Liu JL. CRISPR/Cas9 mediated genome engineering in *Drosophila* . Methods. 2014;69(2):128–36. 10.1016/j.ymeth.2014.02.019 . 24576617

[40] Foster GG, Whitten MJ, Konowalow C, Bedo DG, Maddern RH, Boon DJ. Cytogenetic studies of *Lucilia cuprina dorsalis R*.*-D*. (Diptera: Calliphoridae) Polytene chromosome maps of the autosomes and cytogenetic localization of visible genetic markers. Chromosoma. 1980;81:151–68.

[41] Bedo DG. Differential sex chromosome replication and dosage compensation in polytene trichogen cells of *Lucilia cuprina* (Diptera: Calliphoridae). Chromosoma. 1982;87(1):21–32. 7160277 10.1007/BF00333507

[42] Laverty C, Li F, Belikoff EJ, Scott MJ. Abnormal dosage compensation of reporter genes driven by the *Drosophila* glass multiple reporter (GMR) enhancer-promoter. PLoS ONE. 2011;6(5):e20455 Epub 2011/06/10. 10.1371/journal.pone.0020455 PONE-D-11-06710 [pii]. 21655213 PMC3105068

[43] Arkhipova I, Li J, Meselson M. On the mode of gene-dosage compensation in *Drosophila* . Genetics. 1997;145(3):729–36. 9055082 10.1093/genetics/145.3.729 PMC1207857

[44] Sass H, Meselson M. Dosage compensation of the *Drosophila pseudoobscura Hsp82* gene and the *Drosophila melanogaster Adh* gene at ectopic sites in *D*. *melanogaster* . Proceedings of the National Academy of Sciences of the United States of America. 1991;88(15):6795–9. 1907376 10.1073/pnas.88.15.6795 PMC52175

[45] Bashaw GJ, Baker BS. The *msl-2* dosage compensation gene of *Drosophila* encodes a putative DNA-binding protein whose expression is sex specifically regulated by *Sex-lethal* . Development. 1995;121(10):3245–58. 7588059 10.1242/dev.121.10.3245

[46] Kelley RL, Solovyeva I, Lyman LM, Richman R, Solovyev V, Kuroda MI. Expression of *msl-2* causes assembly of dosage compensation regulators on the X chromosomes and female lethality in *Drosophila* . Cell. 1995;81(6):867–77. 7781064 10.1016/0092-8674(95)90007-1

[47] Bagnall NH, Kotze AC. Evaluation of reference genes for real-time PCR quantification of gene expression in the Australian sheep blowfly, *Lucilia cuprina* . Med Vet Entomol. 2010;24(2):176–81. 10.1111/j.1365-2915.2010.00866.x . 20604863

[48] Altschul SF, Madden TL, Schaffer AA, Zhang J, Zhang Z, Miller W, et al Gapped BLAST and PSI-BLAST: a new generation of protein database search programs. Nucleic Acids Res. 1997;25(17):3389–402. 9254694 10.1093/nar/25.17.3389 PMC146917

[49] Fieller EC. Some problems in interval estimation. Journal of the Royal Statistical Society: Series B. 1954;16(2):175–85.

