# Supplementary material for: Dosage Compensation of X-Linked Muller Element F Genes but Not X-Linked Transgenes in the Australian Sheep Blowfly
Source: PLoS One. 2015 Oct 27;10(10):e0141544. doi: 10.1371/journal.pone.0141544 (PMC4624761; doi:10.1371/journal.pone.0141544)
Supplement: S1 Table — (DOCX) [file pone.0141544.s001.docx]

**S1 Table. Transgenic lines of *Lucilia cuprina* that show X-linked inheritance**

| **Line** | **Marker** | **Additional Gene** | **Reference** |
| --- | --- | --- | --- |
| HS14 | Lch83-ZsGreen | none | 20 |
| FL3-3 | Lch83-ZsGreen | tetO-tTA (FL3) | 21 |
| FL11-1 | Lch83-RFPex | tetO-tTA (FL11) | 21 |
| FL12-9 | Lch83-RFPex | tetO-tTA (FL12) | 21 |
| Slam-5 | Lch83-RFPex | Chslam-turboGFP | 22 |
| EF1-2 | Lch83-RFPex | tetO-Lshid[Ala2] | 23 |
| EF1-10 | Lch83-RFPex | tetO-Lshid[Ala2] | 23 |
| EF3-H | Lch83-RFPex | tetO-Lshid | 23 |
| DR2-11 | Lch83-ZsGreen | Lsbnk-tTA | 23 |
| DR2-18 | Lch83-ZsGreen | Lsbnk-tTA | 23 |
| DR3-9 | Lch83-ZsGreen | Lsspt-tTA | Yan and Scott, unpublished |
| DR3-12 | Lch83-ZsGreen | Lsspt-tTA | Yan and Scott, unpublished |
