# Supplementary material for: Dosage Compensation of X-Linked Muller Element F Genes but Not X-Linked Transgenes in the Australian Sheep Blowfly
Source: PLoS One. 2015 Oct 27;10(10):e0141544. doi: 10.1371/journal.pone.0141544 (PMC4624761; doi:10.1371/journal.pone.0141544)
Supplement: S2 Table — (DOCX) [file pone.0141544.s002.docx]

**S2 Table. Abundance of reference genes in male and female genomic DNA.**

| **Gene** | **Male Mean Ct^a^ ± SEM** | **Female Mean Ct ± SEM** | **M/F Ratio** |
| --- | --- | --- | --- |
| *Lc 28S rRNA* | 14.16 ± 0.038 | 14.010 ± 0.059 | 1.011 |
| *LcGST1* | 23.26 ± 0.061 | 23.185 ± 0.033 | 1.003 |
| *Lc α-tubulin* | 23.44± 0.028 | 23.450 ± 0.035 | 0.999 |

^a.^ Mean Ct of quadruplicate wells
