# Supplementary material for: Dosage Compensation of X-Linked Muller Element F Genes but Not X-Linked Transgenes in the Australian Sheep Blowfly
Source: PLoS One. 2015 Oct 27;10(10):e0141544. doi: 10.1371/journal.pone.0141544 (PMC4624761; doi:10.1371/journal.pone.0141544)
Supplement: S3 Table — (DOCX) [file pone.0141544.s003.docx]

| **Gene** | **Forward Primer** | **Reverse Primer** |
| --- | --- | --- |
| *Lc 28S rRNA* | ACCACTGTTCACACGAAACCCTTC | ATCTCGGTTGGATTTTAAACTTTGAAA |
| *Lc α-tubulin* | GTGATTTGGCCAAGGTACAACGTG | CGACGTACCAGTGGACGAAAGC |
| *Lcaru* | TAGTGGAGGTATGCATGGTCAAAA | GGAATAGCTGTGTTGTTCTTTGCT |
| *LcATPsynβ* | TCAACAACGTTGCCAAGGCCCATGGTG | GGTGATGGAGCCCTTCTTGGTGGTGGT |
| *LcCG1970* | CCTCCTTCCCGTTCCGAAAT | TTGCACCAGGTGGAACTTGA |
| *LcEph* | CACCGGGTCCTCCAACAAAT | ATCGCGGACTGGTGTATTCC |
| *LcEphrin* | CATAGCGACGGGTGGTAGTT | CGTTATGTCCTCGCCGCTAT |
| *LcGST1* | GCCAGTGTCAGCACCTTTG | GCAACCTTCCCAGTTTTCATC |
| *Lcgw* | AACAATGGTACGGACTTATGGGAA | AAGTGCCACCCAAATTTGTAGATG |
| *Lchsp83* | GGTCATCCGCAAGAACTTGGTCA | AGCAGAGGTGTGTGGAAACGCAAG |
| *LcJwa* | AGCAACATATACCCGATTAAAAGCG | TGTCATCGCTATAATTTTGCACCTC |
| *LcSlip1* | AGTTCGAAGTGCTATAACCACACT | TAAGCACTGGATGAATTGTCCTGA |
| *LcThd1* | CCTTCTCCAGCACCATGTGA | GTGCCACCAGCTCTAACGAT |
| *Lczfh2* | CAGAGGAGTCAGCTGCTGTATTAA | CTTGACACAAAGGACACTGTACAC |
| *RFPex* | CTTGCCATTCGCCTGGGATA | TCGGGAATATCAGCGGGATG |
| *ZsGreen* | ACCACACACAGATTAATAGCCTGT | GTCAAAGCACGGTCTAACAAAAGA |

**S3 Table. Primers utilized for qPCR.**
